# Superolateral medial forebrain bundle deep brain stimulation in major depression: a gateway trial

**DOI:** 10.1038/s41386-019-0369-9

**Published:** 2019-03-13

**Authors:** Volker A. Coenen, Bettina H. Bewernick, Sarah Kayser, Hannah Kilian, Jan Boström, Susanne Greschus, René Hurlemann, Margaretha Eva Klein, Susanne Spanier, Bastian Sajonz, Horst Urbach, Thomas E. Schlaepfer

**Affiliations:** 1 Department of Stereotactic and Functional Neurosurgery, University Hospital Freiburg, Freiburg, Germany; 2 Faculty of Medicine, University of Freiburg, Freiburg, Germany; 3 Department of Neurosurgery, University Hospital Bonn, Bonn, Germany; 4 Department of Psychiatry and Psychotherapy, University Hospital Bonn, Bonn, Germany; 5 Department of Interventional Biological Psychiatry, University Hospital Freiburg, Freiburg, Germany; 6 Division of Neuroradiology, Department of Radiology, University of Bonn, Bonn, Germany; 7 Department of Neuroradiology, University Hospital Freiburg, Freiburg, Germany; 8 Departments of Psychiatry and Mental Health, The Johns Hopkins University, Baltimore, MD USA

**Keywords:** Target validation, Drug development

## Abstract

Short- and long-term antidepressant effects of deep brain stimulation (DBS) in treatment-resistant depression (TRD) have been demonstrated for several brain targets in open-label studies. For two stimulation targets, pivotal randomized trials have been conducted; both failed a futility analysis. We assessed efficacy and safety of DBS of the supero-lateral branch of the medial forebrain bundle (slMFB) in a small Phase I clinical study with a randomized-controlled onset of stimulation in order to obtain data for the planning of a large RCT. Sixteen patients suffering from TRD received DBS of the slMFB and were randomized to sham or real stimulation for the duration of 2 months after implantation. Primary outcome measure was mean reduction in Montgomery–Åsberg Depression Rating Scale (MADRS) during 12 months of DBS (timeline analysis). Secondary outcomes were the difference in several clinical measures between sham and real stimulation at 8 weeks and during stimulation phases. MADRS ratings decreased significantly from 29.6 (SD +/− 4) at baseline to 12.9 (SD +/− 9) during 12 months of DBS (mean MADRS, *n* = 16). All patients reached the response criterion, most patients (*n* = 10) responded within a week; 50% of patients were classified as remitters after 1 year of stimulation. The most frequent side effect was transient strabismus. Both groups (active/sham) demonstrated an antidepressant micro-lesioning effect but patients had an additional antidepressant effect after initiation of stimulation. Both rapid onset and stability of the antidepressant effects of slMFB-DBS were demonstrated as in our previous pilot study. Given recent experiences from pivotal trials in DBS for MDD, we believe that slow, careful, and adaptive study development is germane. After our exploratory study and a large-scale study, we conducted this gateway trial in order to better inform planning of the latter. Important aspects for the planning of RCTs in the field of DBS for severe and chronic diseases are discussed including meaningful phases of intra-individual and between-group comparisons and timeline instead of single endpoint analyses.

## Introduction

Most patients suffering from major depressive disorder (MDD) respond to a combination of psychotherapy and pharmacotherapy [1]; however, about 20–30% of MDD patients fail to respond to established treatments [2] and are therefore classified as suffering from treatment-resistant major depression (TRD). Deep brain stimulation (DBS) has provided therapeutic benefits for otherwise treatment-resistant disorders [3] and has emerged as a potential treatment option for severe TRD.

Several open-label pilot studies have documented significant short- and long-term antidepressant effects of DBS of the subgenual cingulate gyrus (cg_25_) [4], the ventral capsule and ventral striatum (vc/vs) [5, 6], and the nucleus accumbens (NAC) [7–9].

Results from randomized-controlled trials (RCTs) are inconclusive: two company-sponsored studies stimulating vc/vs [10] and cg_25_ [11] failed to show superiority of DBS to sham stimulation at short time; they had to be terminated after a previously planned futility analysis in a subgroup of planned patients [12]. On the contrary, superior effects of DBS vs. sham stimulation have been demonstrated in a more adaptive, individualized study design [13]. Thus traditional study designs with short times for parameter optimization,  single endpoints and a sham condition directly after implantation seem inadequate for the assessment of antidepressant effects of DBS in TRD as a chronic, severe medical condition.

The supero-lateral branch of the medial forebrain bundle (slMFB) was proposed as a novel DBS target [14, 15] based on its key function within the human reward system and its putative dysfunction in TRD [16]. The clinical validity of stimulation at this target is supported by both findings of early-onset antidepressant action and a response rate of 85% after 3 months of treatment [17, 18]. We demonstrated antidepressant efficacy to be sustained for >4 years; most importantly, responders maintained the response criterion in the very long term [19]. These results have been replicated independently recently [20]. Discontinuation of stimulation seems to cause reoccurrence of symptoms [21], a clear indication of efficacy of stimulation. Taken together, these findings make the slMFB a very promising target for the treatment of TRD [9].

This study aimed (1) to assess long-term efficacy and safety of DBS of the slMFB in a gateway study design and (2) to evaluate the feasibility and the optimal timing of a sham condition (2 months) for the planning of a larger RCT.

## Methods

### Patients

Sixteen patients received slMFB DBS for 12 months; all patients provided written informed consent. At baseline, all patients suffered from severe TRD according to Diagnostic and Statistical Manual of Mental Disorders (DSM)-IV [Structured Clinical Interview for DSM-I and II] [22]. One patient with bipolar depression was also included in this study (see eTable 4). Three raters analyzed clinical records. Inclusion criteria were a minimum score of 21 on the 24-item Hamilton Depression Rating Scale (HDRS_24_) [23] and a score <45 in the global assessment of functioning (GAF) [24] (see [18] for inclusion criteria). Medication was kept constant for at least 8 weeks before and after surgery. The antidepressant treatment history form (ATHF) score [25] for the current depressive episode was at least 3, defining a treatment resistance for the current antidepressant treatments for all patients. A score of “3” is the threshold for considering a trial adequate and the patient resistant to that treatment [25]. Common screening failures were comorbid psychiatric disorders (e.g., substance dependency, schizoaffective disorder, posttraumatic stress disorder, severe personality disorder) or surgical contraindication. The study was performed between January 2013 and February 2016. All patients were diagnosed as having severe TRD with an ATHF score of at least 3 in the current episode.

### Study design and outcome measures

The study was planned and implemented as a Phase I clinical single-center trial conducted according to Good Clinical Practice guidelines. A double-blind (clinical rater and patient) randomized-control (DBS active vs. sham) condition was implemented for 8 weeks after surgery. The Institutional Review Board of the University of Bonn approved of this study; the protocol is registered with http://Clinicaltrials.gov with the identifier NCT01778790.

Psychiatric assessments were conducted weekly for the first 17 weeks after surgery, then biweekly until week 23, then every 4 weeks up to 12 months (primary study endpoint). Raters and patients were blinded only during the first 8 weeks after which all patients were actively stimulated.

The **primary outcome measure** was the average reduction in the Montgomery–Åsberg Depression Rating Scale (MADRS) [26] during 12 months of DBS treatment (period of time) as compared to baseline (long-term efficacy measure).

**Secondary outcome measures** included the 28-item HDRS_28_ [23], Beck Depression Inventory (BDI) [27], the short-form of health survey questionnaire (SF-36) [28], evaluating a patient’s subjective change in quality of life, and GAF [24] for 12 months of DBS compared to baseline (long-term efficacy).

Further secondary outcome measure was the difference in the average response on the above-mentioned scales between the DBS group (group A, immediate stimulation) and the sham group (group B, delayed stimulation) during 8 weeks. This randomized-control phase was introduced to understand the effects of surgery (e.g., micro-lesioning effect) or possible placebo response and to assess whether the length and placement of a sham condition immediately after surgery is reasonable.

Safety and tolerability of 12 months of slMFB DBS were also assessed. Safety of the treatment method was documented in a standardized way to the Food and Drug Administration definitions [29]. The Compendium of Neuropsychological Tests [30] was used to assess the level of performance in the following cognitive domains: learning and memory, language, attention, visual perception, and executive function.

Before inclusion, the score of the ATHF [25] was computed. A score of “3” is the threshold for considering a trial adequate and the patient resistant to that treatment. A 50% reduction of depressive symptom severity in MADRS was classified as response, while a MADRS score <10 was classified as remission according to broadly accepted conventions in depression research [18].

### Interventions

**Fig. 1:**
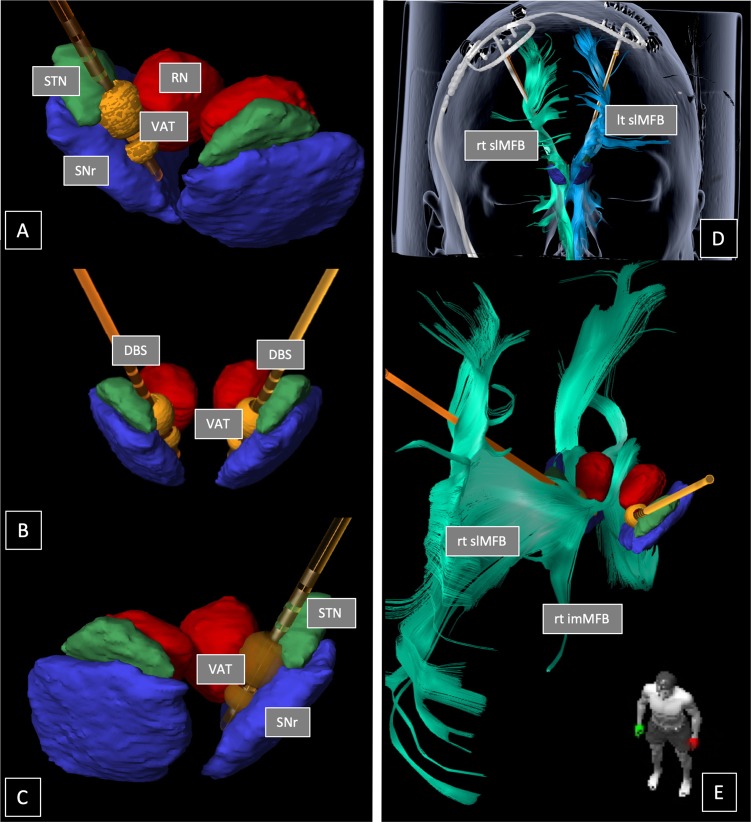
Reconstruction of electrode position for Patient H (responder) including volume of tissue activated (VAT; dumbbell-shaped, orange) simulation in bipolar mode (3 mA, 60 µs, 130 Hz, 1+, 2−, 3−). **a** View of right deep brain stimulation (DBS) electrode positioned between substantia nigra (SNr) and red nucleus (RN). Note how VAT is located in the cleft space (white matter) and barely touches the surrounding structures like the subthalamic nucleus (STN). **b** View from anterior. **c** View of left DBS electrode. **d**, **e** DBS electrodes located inside the left (lt, blue) and right (rt, green) superolateral medial forebrain bundle (slMFB), respectively. Original image data reconstructed with the Elements ® (BrainLab, Munich, Germany) stereotactic planning software. VAT simulation was performed with Guide XT (Boston Scientific, CA, USA). The electrode is octopolar (for the sake of presentation), whereas in the trial quadripolar electrodes were used. Geometries are identical

Stereotactic surgery: A detailed description of slMFB DBS surgery was recently published [18]. In brief, bilateral DBS electrodes (model 3389, Medtronic, USA) were implanted with the patient under local anesthesia (NexFrame, Medtronic, USA; or Leksell G-Frame, Elekta, Sweden). Techniques of Diffusion Tensor Imaging-assisted neuronal circuit DBS (StealthViz DTI, Medtronic, USA) were applied as already described in our previous publication [18]. After fiber-tractographic reconstruction of the slMFB and targeting the slMFB (StealthViz DTI, Medtronic USA) [14], microelectrode recording (FHC MME, FHC Bowdoin, USA) was used to identify the target located medial to the subthalamic nucleus (STN) and the substantia nigra (cf. Fig. 1). Intraoperative test stimulation was utilized to see acute antidepressant effects (specific for single-side stimulation) and to identify the typical unilateral oculomotor activation (see Discussion for detail) and a typical heart rate variation as side effects.

In this study, we have intraoperatively looked for psychotropic effects that might possibly occur [31]. Euphoria, mirthful laughter, confusion, etc., typical psychiatric effects under STN DBS in Parkinson’s disease [32] have not been observed neither during surgical placement of electrodes and test stimulation nor in the context of chronic adjustment of stimulation parameters. We have occasionally seen some unilateral and mild *aversive* response during test stimulation (patients never mentioned “anxiety” but “aversiveness” on request) on more posterior electrode positions. If this occurred intraoperatively, we immediately changed to a different (typically more anterior) position. Subsequently, this effect resolved. We have never seen these effects during initiation of chronic stimulation nor during the chronic stimulation phase itself.

Summing up, the key points of the intraoperative identification and implantation of the slMFB are: (1) diffusion tensor imaging (DTI) tractographic depiction of the slMFB, (2) microelectrode recording to exclude nuclear environment (STN, substantia nigra, red nucleus) from stimulation, (3) intraoperative test stimulation showing (a) autonomous response (heart rate increase), (b) appetitive motivation response, and (c) the threshold for oculomotor effects. Correct intraoperative identification of slMFB is determined with postoperative helical computed tomography (CT). We have further explained in detail in the supplement section how we used microelectrode-recording with three parallel electrodes to make sure that surrounding structures (like the STN) are excluded from stimulation.

### Blinding phase

After surgery, patients were randomized into two groups (sham vs. stimulation). The stimulation group received immediately stimulation at the next visit; the sham group did not receive stimulation for the next 8 weeks. After 8 weeks, the stimulation was also initiated in the sham group. Patients and raters were blinded for the group. The device was checked on each visit for both groups, suggesting a possible parameter change. The time spent at each visit, controlling the device, was kept constant between groups. Patients were asked randomly what condition they believed to belong to.

### Stimulation

Electrode contact selection and titration of stimulation was described before [18]. See supplementary material for more details.

### Statistical analysis

All analyses were performed as intent-to-treat (ITT) analyses with last observation carried forward method to prevent overestimation of the antidepressant effect.

Outcome measures (12 months of slMFB-DBS, primary study endpoint) are compared with baseline measures and analyzed with a General Linear Mixed Models (GLMM) approach. For between-group comparisons (8 weeks sham vs. stimulation), we also used a GLMM approach. To control for the effect of baseline characteristics, baseline score was included in all analyses. GLMM was also used to assess if group B (sham) had an additional antidepressant response after initiation of stimulation.

The number of responders and remitters was calculated for each month and the number of weeks of stimulation to reach first response is given. Between-group differences in demographic and clinical characteristics at baseline were tested with Student’s *t* test for independent samples.

## Results

### Study population

We screened 300 patients with TRD for eligibility and included 16 of these patients in the study between 29 and 71 years of age (mean ± SD: 51.6 ± 10.2 years) with a current depressive episode of 10.3 years duration in average (±9.2) (see eTable 4 for demographic and clinical details).

At study entry, patients had received treated on average with 18.9 (10.3) antidepressant medications, had received on average 20 electroconvulsive therapies, and on average 70 h of  psychotherapy without response.

### Dropouts/early termination

Two patients did not complete the full study protocol: one patient was excluded in month 4 from the study due to continued methylphenidate misuse (180 mg/day) and non-compliance with the study protocol; one patient left the study due to physical abuse by her alcoholic partner after month 7. Two patients had infections at the implanted pulse generator (IPG) implantation site and one had to have revision surgery with a relocation of the IPG but was not excluded from the study, see Fig. 1 for consort study flow chart.

### Stimulation parameters

Patients were stimulated initially with 2.1 mA in average (SD: 0.5 mA) and three of the four contacts were activated (bipolar setting: one anodal, two cathodal contacts above, see supplementary material). Mean stimulation amplitude throughout the whole 12 months of stimulation was 3.0 mA (SD: 0.5 mA). Induction of side effects of medial STN stimulation like disorientation, depression, etc., were not observed. DBS electrodes (model 3389, Medtronic, USA) were implanted as to typically reach the deepest part of slMFB with the electrode tip and on the same day were connected to an internal pulse generator (ACTIVA PC, Medtronic, USA; located subcutaneously in the abdominal region) in a separate session under general anesthesia. CT data were fused to planning data in order to check the achieved electrode positions. All electrodes reached the slMFB.

### Efficacy

#### Response at primary study endpoint (DBS during 12 months)

**Fig. 2:**
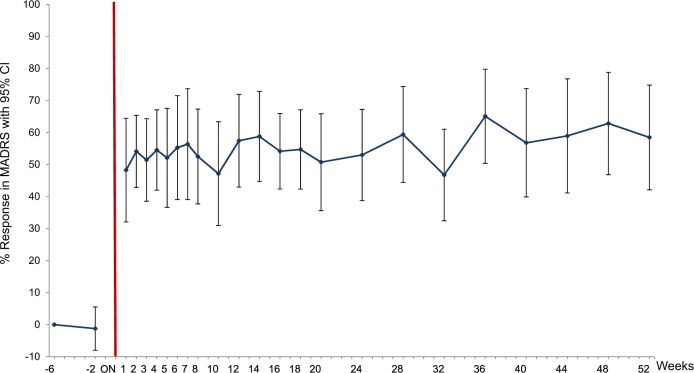
Long-term improvement in depression during deep brain stimulation

There was a significant decrease in average MADRS from 29.6 (SD +/− 4) at baseline to 12.9 (SD +/− 9), mean MADRS during 12 months of DBS, whole group analysis, *n* = 16, ITT, GLMM: Factor GROUP; *p* < 0.0001; df = 15; *t* value −7.28) (see eTable 3). All patients reached response status during the study. On average, patients reached response during 61% of months they participated in the study (see Fig. 2).

At month 12 after DBS initiation (single time point), 8 of the 16 patients (50%) were classified as remitters (MADRS ≤ 10).

### Sham vs. real DBS

The study groups did not differ with regard to demographic (age, sex, duration of education) or clinical characteristics (ATHF Score, lengths of current episode, age at onset, suicide attempts) at baseline (see eTable 4).

### Time to response

The mean time for first response was 1 week in the majority of patients (*n* = 10): 2 patients responded within 2 weeks, 1 patient within 3 weeks, 1 patient within 5 weeks, 1 patient within 10 weeks, 1 patient within 28 weeks.

### Feasibility of sham condition

**Fig. 3:**
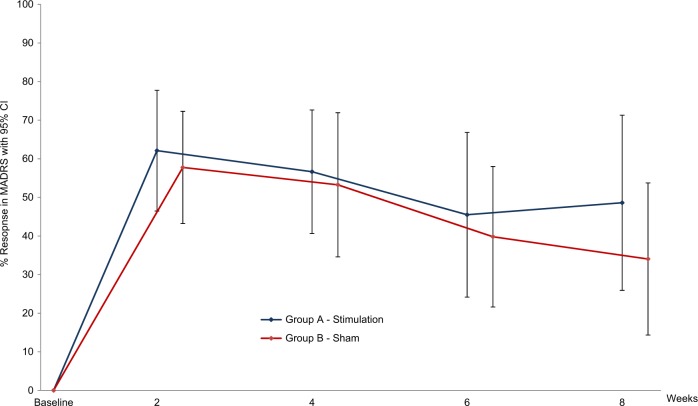
Improvement of depression: active deep brain stimulation vs. sham

All patients have been asked about what they believed regarding which group they had been assigned to in the first, sham controlled phase of the study. Overall, patients had a chance probability to guess their assignment, neither patients nor raters were aware as assessed with regular interviews. Interestingly, a single patient belonging to the sham group had a strong amelioration of symptoms and therefore was convinced to be in the stimulated group, whereas one patient only from the stimulated group did not have an immediate antidepressant effect and therefore was convinced to belong to the sham condition. There was a sizable setting effect in the sham group which led to the fact that effects in both groups could not be differentiated in the relatively short (8 weeks) blinded phase of the study (Fig. 3).

### Cognition

No difference in cognitive domains was found between groups (sham vs. active stimulation) after 8 weeks (see eTable 1 supplementary material). In most cognitive domains, there were no statistical differences between baseline performance and 6 or 12 months of DBS in the whole group; however, verbal learning (VLMT) and language IQ (MWT) significantly improved between baseline and 12 months (see eTable 2 supplementary material).

### Secondary outcomes and response during the course of study (each month)

On average, MADRS and HDRS scores were significantly reduced during DBS compared to baseline in the whole sample (see eTable 3 and Fig. 2).

Quality of life (mental health, SF-36mh) was improved significantly through most months when stimulated with DBS and was augmented about 100%. Physical health was not improved significantly. The level of functioning (GAF mean) changed significantly from 40.8 (“serious impairment in social, occupational, or school functioning”) at baseline to 74.2 (“no more than slight impairment”). Subjective patients’ ratings of depression (BDI) were reduced significantly in all months (except month 8 (see eTable 3).

### Adverse events

Common adverse events were as in previous studies of DBS to the same target oculomotor symptoms (blurred vision, and double vision), which in every single instance could be resolved by parameter changes, especially by adjusting the stimulation amplitude (see Table 1). Oculomotor side effects typically limited the raise in amplitude at the lowest contact. Some patients adapted to symptoms of strabismus after several hours when the amplitude was increased, but most patients’ stimulation settings were optimized without inducing any side effects. There was a single stimulation change-induced instance of clinical and transient hypomania that was not further quantified in one patient (1/16) only. The episode lasted 3 days without any clinical symptoms of mania. Hypomania is not a significant side effect of slMFB DBS. Nevertheless, hypomania—if undetected—is a serious event and should be closely monitored for. In our case, it resolved after re-programming. Other side effects of stimulation were restlessness in one patient and transient slurred speech in one patient. Furthermore, one patient suffered from hyperkinesia (probably due to inadvertent co-stimulation of the STN), one patient attempted suicide, and one patient misused methylphenidate.

**Table 1:** Adverse events

	Patients	Number of events
Serious adverse events		
Hyperkinesia^a^	1	1
Wound healing disorder, skin irritation leading to the explantation of the IPG	2	3
Suicide attempt^d^	1	1
Drug abuse^d^	1	1
Adverse events		
Vision disorder (blurred vision, strabismus)^a^	16	250
Hypomania^a^	1	1
Restlessness^a^	2	2
Tumble^d^	3	3
Pain at IPG and scar^b^	1	1
Disequilibrium^a^	2	2
Increased blood pressure^d^	4	4
Tachycardia^d^	1	
Dyspnoea^d^	1	1
Gastrointestinal disease^d^	6	8
Back pain	1	10
Abdominal pain^d^	1	1
Headache^d^	1	1
Influenza^d^	1	1
Bronchitis^d^	2	2
Hypothyroidism^d^	2	1
Abscess at the injection site of diabetes treatment	1	3
Rheumatism (soft part)	1	1
Transaminase increase	1	1
Speech disorder (blurred speech)	1	2

Adverse events and serious adverse events up to primary study endpoint (12 months). For the first patient, zopiclone was stopped at week 24 and quetiapine was stopped at week 38, because of improvement in depression. For the second patient, zopiclone was stopped at week 12, mianserine at week 25, and agomelatine was reduced from 50 to 25 mg at week 46, again because of improvement of depression symptoms. One patient was not compliant to medication and stopped all medications in month 2

*IPG* implanted pulse generator

^a^Associated with stimulation/parameter change

^b^Surgery related, successfully treated with antibiotics

^c^Device malfunction

^d^Not related to the study

Severe wound healing disturbances led to two surgical revisions (later re-implantation) of the IPG in one patient. Another patient developed atrophic wound healing problem in the region behind the ear (cable) and at that time elected for removal of the system. No other serious adverse events were observed.

During the observational period of 1 year, 14 patients received changes to their antidepressant medication (24 times antidepressants stopped; 34 times antidepressants were started).

## Discussion

This study aimed (1) to assess long-term efficacy and safety of DBS of the slMFB and (2) to evaluate the feasibility and the optimal timing of a sham-controlled condition for this new target. In a previous pilot study, rapid and sizable antidepressant response of this form of DBS has been demonstrated [18] and, recently, very stable long-term efficacy (4 years) in the same patient group [19]. We designed this trial as a gateway study with a similar design but with twice the number of patients as in the pilot study on the transition to a truly pivotal study. We believe that this careful and admittedly slow approach will lead to a more robust design of future studies of this costly experimental treatment. It might well be that the comparatively quick development of pivotal studies for two other stimulation targets contributed to the negative results [12].

### Antidepressant efficacy of slMFB DBS

In this study, we replicated rapid, sizeable, and long-term antidepressant efficacy of DBS of the slMFB.

The size of acute effects within days is comparable to our previous results [18] and results of an independent replication [17]. In addition to antidepressant efficacy, a significant increase in quality of life and global functioning measures was observed. Long-term stability of the antidepressant effect over at least 4 years stimulating the slMFB has been published as well as a normalization in quality of life and global functioning [19]. We found a benign efficacy to side effect profile that—from a safety standpoint—is comparable to previous DBS studies in TRD. Transient oculomotor effects (strabismus) are idiosyncratic for stimulation of the slMFB target because of its close topographical vicinity to the origin of the oculomotor nerve [18]. Cognition remained unchanged besides a minor increase in measures of verbal learning.

The fast time to response (1 week), the high proportion of responders (100% of patients were responders at least 1 month during the study), the stability of response (60.4% of months in response in average) as well as the sizeable reduction of depression severity render the slMFB a promising stimulation target for DBS in TRD.

Significant antidepressant effects of DBS at several targets [6, 7, 18, 33] have been demonstrated in open-label studies. Two industry-sponsored sham-controlled trials stimulating vc/vs (ventral capsule and ventral striatum) [10] and cg_25_ (Brodman’s area 25 or subgenual cingulate gyrus) [11, 34] were terminated due to the results of interim futility analyses of small proportions of patients intended to treat. Both studies were not adequately designed to prove the superiority of DBS compared to sham stimulation [12, 35]. A third study has demonstrated superiority of DBS to sham stimulation stimulating vc/vs in a more adaptive design [13].

Suboptimal timing of the sham condition, putative placebo and micro-lesioning effects, an insufficient time for parameter optimization as well as suboptimal surgical targeting [36, 37] are possible explanations for these data [10, 11]. As we have learned from studies on the antidepressant effects of vagus nerve stimulation, the peak effect of a treatment might be observable at a later time point than previously expected [38]. It has been demonstrated [13] that parameter optimization for several months could be necessary in DBS to some targets. Therefore, in this Phase I clinical trial we decided to analyze the timeline of the clinical effect, the time needed for parameter optimization, and the feasibility of a placebo group in a small sample before planning a larger RCT.

### Acute antidepressant effects after surgery

We observed a strong acute antidepressant response in most stimulated patients within 1 week; a similar effect occurred in the sham stimulation group. This is in line with data from an independent replication study [17] that also reported an acute effect before stimulation onset over 4 weeks in their sample stimulated at the slMFB. The most likely explanations for this pattern are (1) micro-lesioning effects or (2) placebo effects.

In studies of Parkinson’s disease, an acute amelioration of symptoms has been described as “micro-lesioning effect” before the onset of stimulation [39]. For most movement disorder DBS surgery, micro-lesioning effects are typical and are reflective of future stimulation efficacy. During DBS electrode insertion in the present study, we have seen that patients felt an acute amelioration of symptoms [31]. Possibly, electrode insertion at the slMFB might lead to transient silencing of phasic dopaminergic neurons in the ventral tegmental area (VTA), which in rodents are known to cause an increased susceptibility for stress [40]. Regarding micro-lesioning effects, DBS has been demonstrated to induce neuro-inflammation at the target site in rats that can be blocked with anti-inflammatory drugs [41]. In an analysis of clinical data in TRD DBS patients from the same group, an acute antidepressant effect was reduced in those patients taking anti-inflammatory medication after surgery [41].

Placebo effects are more probable at the beginning of an intervention and larger in more invasive interventions [42]. The conviction of the patient to belong to a certain interventional group and the study design also seem to have an influence on patient’s expectations [42]. In our study, sham stimulation effects could therefore possibly contribute to the acute effects seen in both groups. On the other hand, patients with TRD are less prone to develop placebo effects [43]. Because of a history of non-response to many antidepressant treatments, patients may not expect an antidepressant effect of further treatments. In addition, any putative placebo effect would likely explain short-term effects but not long-term antidepressant effects as detected in our study. However, it is impossible to rule out a placebo response as the result of the intense study interactions in these patients.

The introduction of a sham stimulation phase in the study directly after surgery seems critical, because parameters are not optimized and several confounding factors (placebo expectation, micro-lesioning effect) might severely influence efficacy. To our knowledge, there is only one study that has documented, in 16 patients, that a placebo phase located later during the study timeline, including the termination of DBS in patients after an individualized parameter optimization phase around 6 months, produced significant between-group effects [13]. Interestingly, most patients had to be “rescued” within days after DBS termination because of a strong worsening of symptoms. In our study, we did not include a condition with DBS termination, but several patients from the first [19] and the present study had an unforeseen, double-blind stimulation interruption (e.g., due to battery depletion). This has led to an immediate worsening of symptoms and in one case even to a relapse in depression [21].

### Surgical considerations

The slMFB as region for chronic high frequency stimulation in TRD was introduced as the first target utilizing the DTI tractographic approach for (a) scientific rationale, (b) general and individual target identification, and (c) stereotactic planning obeying the overall concept of a modulation of network hubs with the DBS technology [18, 44, 45].

The oculomotor nerve (CNIII) traverses the lateral pigmented nucleus (inside the midbrain) as part of the VTA. CNIII marks the entry into the lateral part of the VTA. Thresholds <1.5 mA lead to withdrawal and more superficial positioning of the electrode after repeated testing. This allows to stimulate the more superficially located slMFB with high enough current amplitude. The bipolar stimulation (cf. Fig. 1) makes CNIII activation during chronic stimulation less likely. CNIII is easily activated with stimulation but anatomically runs almost perpendicular with respect to our electrode’s trajectory. Bipolar stimulation creates an electric field parallel to the electrode [46] and parallel to the slMFB and steers current away from CNIII. Nevertheless, oculomotor activation during stimulation in our eyes is the hallmark for antidepressant response and a parameter to keep stimulation close to the VTA in the slMFB (for more details, see [47]).

A thorough analysis of the surgical technology, including techniques applied in this trial, has been published recently [45]. In the light of our initial results [18, 19], others have started to apply similar approaches of tractographic imaging to improve targeting and to optimize antidepressant efficacy in a region that otherwise is inherently silent for acute stimulation (side) effects (cg_25_) [36, 37]. Advanced imaging technology (DTI) in combination with micro-electrode recording and immediately visible side effects (strabismus) and autonomous effects (heart rate variation) upon macro-stimulation facilitate intraoperative identification of the slMFB target region and help to improve electrode placement and stimulation efficacy. In this respect, slMFB DBS—unlike other target regions for TRD—shares many features of movement disorder surgery (e.g., Parkinson’s disease, dystonia, etc.) and might therefore prove to be advantageous.

### Stimulation of the slMFB

We have recently performed several analyses including an extensive VTA analysis. This is the focus of ongoing research, and at this moment, it would be outside the scope of this paper because of the complexity of the data. In a recent publication, we addressed the surgical technique [47]. *In this publication, all the active contacts of this trial were visualized and could be evaluated*. We argued that stimulation contacts correlated with response were all located inside the triangle (white matter) between STN/substantia nigra reticulata (SNR), red nucleus, and mammillothalamic tract. There was no preference for effective contacts to be located lateral toward the STN/SNR. Also, in a midcommissural point analysis (coordinates) the responders/non-responders are almost evenly distributed over the region with no preference for the STN region [47]. White matter has a much lower activation threshold than gray matter. Moreover, the heavy anisotropy which surrounds a contact that is located in white matter stops the electric field to expand far away from the electrode. These facts are typically not represented in today’s VTA analyses, which all heavily and provenly overestimate the size of the effectively stimulated tissue [48]. At the same time, recent work shows that the stimulation activates an axonal structure best, when field lines are rather parallel to the fiber tract of action [46]. This is the case in our bipolar stimulation, which is performed in slMFB DBS patients (cf. Fig. 1). White matter-specific VTA modeling is needed to shed more light on this issue. Clinically, we have seen effects that are reminiscent of STN stimulation (dyskinesia) only occasionally, but other effects like the “appetitive motivation response” is not seen in any other target regions in proximity to the stimulated region. However, we cannot completely rule out a certain sum effect from co-activation of medial STN or medial STN tributaries to the slMFB [45].

### Trial design and sham conditions in DBS for TRD

TRD is a chronic, severe disease and DBS is a long-term treatment method. One should be aware that classical designs from pharmacological studies (a single, primary endpoint after 3 months, between-group comparison) seem not adequate to assess efficacy; instead, more adaptive, individualized study designs are required.

**Fig. 4:**
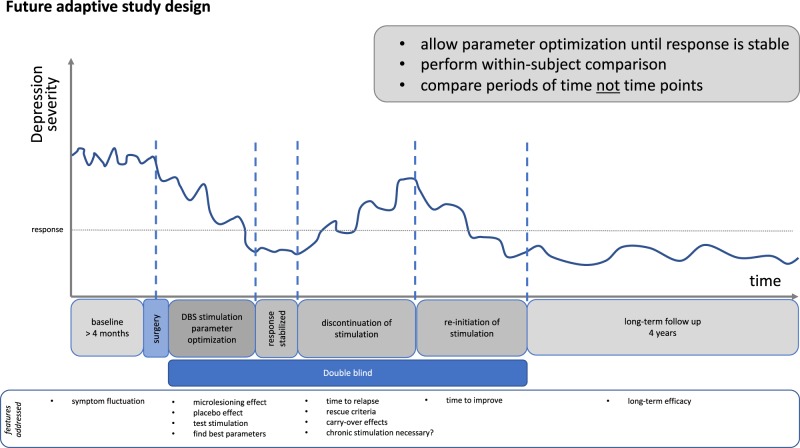
Study design for deep brain stimulation studies in treatment-resistant major depression

It is debatable whether between-group comparisons represent an adequate methodology for assessing clinical efficacy in DBS trials for TRD. Adequate comparison groups are per definition not easily available as long as we only include high-level TRD patients. As an advantage, DBS allows the intra-individual comparison of double-blind stimulation and sham phases along the course of the treatment. We have also demonstrated that patients are not aware of their stimulation condition (sham vs. active DBS) during placebo phase in this study. Thus a study design comparing DBS phases to placebo phases in the whole group after the optimization of stimulation parameters could be more adequate for this intervention and patient population (see Fig. 4 for an example trial design using the intra-individual comparison of phases with DBS and with sham).

### Limitations

This is the first Phase I clinical study including a randomized sham-control phase in DBS of the slMFB, but the small sample size limits the interpretation of results. The high percentage of responders in the first study [18, 19] and lacking knowledge about the micro-lesioning effect and other confounders after surgery have certainly led to an overestimation of effect size for the planning of this study. A longer and differently placed placebo phase might have also demonstrated more pronounced between-group effects. However, the local ethics committee found a >8 weeks sham period not acceptable.

## Conclusions

DBS of the slMFB has demonstrated acute as well as long-term antidepressant effects in patients suffering from TRD. The surgical procedure of slMFB DBS has many features of movement disorder surgery (imaging, electrophysiological identification, test stimulation) and the target region is identifiable during surgery, which might be advantageous in comparison to the other target regions. No severe side effects related to the stimulation were observed. Quality of life and social functioning significantly improved. Acute antidepressant effects were observed also without stimulation after surgery, possibly as a response to the electrode insertion—which might be indicative for a better future response—or placebo effects. These effects need to be studied in more detail and should be considered in the planning of larger RCTs. Our study points to the fact that different study designs are needed for different DBS stimulation targets—even in the same disease—and that target-specific time courses of response have to be reflected in the planning phase. In addition, the present analysis, considering the response at all time points, seems to be more adequate for this kind of interventions.

## Funding and disclosure

This investigator-initiated study was funded in part (DBS device, battery exchange, medical costs, and limited support for study nurse) by a grant of Medtronic Inc. to TES and VAC. All other authors state no conflict of interest. The sponsor had no influence on design and conduct of the study; collection, management, analysis, and interpretation of the data; and preparation, review, or approval of the manuscript.

## Supplementary information

Supplementary Online Material

CONSORT Flowchart

eTable 1

eTable 2

eTable 3

eTable 4
