# Supplementary material for: Superolateral medial forebrain bundle deep brain stimulation in major depression: a gateway trial
Source: Neuropsychopharmacology. 2019 Mar 13;44(7):1224–32. doi: 10.1038/s41386-019-0369-9 (PMC6785007; doi:10.1038/s41386-019-0369-9)
Supplement: Supplementary file 1 — Supplementary Online Material [file 41386_2019_369_MOESM1_ESM.docx]

**Supplementary Online Material**

**Study Design**

Adherence to inclusion criteria as stated in the protocol was reviewed by an external psychiatrist who is experienced in TRD. Informed consent was obtained from all patients. Data management, 100% monitoring, statistical analyses and randomization was performed by the Center for clinical studies at the University Hospital Bonn.

**Surgical Technique**

Use of two navigated/stereotactic frames (Nexframe, Medtronic, USA / Leksell Frame, Elekta, Sweden): In the first trial we used the NexFrame system (Medtronic, USA) which was the standard of our service at this time. After convincing ourselves (in clinical Parkinsons’s STN cases) that this head-mounted frame can perform badly with respect to the deliverance of accuracy we changed the implantation procedure to a standard stereotactic frame type implantation. In the postoperative analysis we could not find differences in the electrode placements between the systems.

Microelectrode recording (MER): A priori criteria were the identification of the trajectory with the least electrophysiological activity. The STN/SNr are typically seen on the lateral trajectory. Typically, anterior and central trajectories should be silent. Occasionally we see the red nucleus on the central trajectory and then go for test stimulation on the anterior path. Details are the topic of a separate publication.

Acute and intraoperative stimulation effects: The intraoperative oculomotor effects are important to understand the implantation depth (z-axis) of the implantation. At the deepest point of the implantation an amplitude of 1.5mA (0.5 mA increments tested) should be possible to allow for an efficacious stimulation in the contacts more superficial (thus located in the middle of the bundle). If oculomotor effects occurred too soon (<1.5mA) the electrode tip was withdrawn (more superficial implantation). An intraoperative acute effect was seen in nearly all cases on the first trajectory. Occasionally we chose to go to a more anterior trajectory if an acute effect could not be elicited. The details are topic of a separate publication which is currently in preparation.

**Stimulation**

Contact selection and titration of stimulation was described before [^19^](#_ENREF_19). In brief: Bipolar stimulation was initiated with the anodal (positive) contact (typically the second-last deep contact; contact no. 1) below one or two cathodal (negative) contacts (typically contacts no. 2 and 3). Stimulation was always performed bilaterally regardless of results of intraoperative test stimulation. A constant voltage stimulation was initiated (3-5 V; 60us; 130Hz; 24/7) and stimulation was performed with a therapeutic target current of 2.5-3 mA depending on oculomotor side effects that in some cases led to a selection of more superficial contacts.

Stimulation parameters were adjusted over time if the resistance (Ohm) had changed compared to the previous visit or if the antidepressant effect was smaller and if side-effects occurred (see AE table).

**Legends for supplemental figures and tables:**

**eFigure1:** Consort Flow Chart

**eTable 1:** Neuropsychological assessment at baseline and eight Weeks after surgery for each group

**eTable 2:** Neuropsychological assessment at 6 months and after 12 months of DBS stimulation compared to baseline

**eTable 3:** Clinical measures for sham and active DB stimulation

| ***eTable 4a:*** Demographic and clinical characteristics of patients, Group A direct stimulation sham group  ***eTable 4b:*** Demographic and clinical characteristics of patients continued, Group B sham group |
| --- |
