# Supplementary figures and images for: Superolateral medial forebrain bundle deep brain stimulation in major depression: a gateway trial
Source: Neuropsychopharmacology. 2019 Mar 13;44(7):1224–32. doi: 10.1038/s41386-019-0369-9 (PMC6785007; doi:10.1038/s41386-019-0369-9)

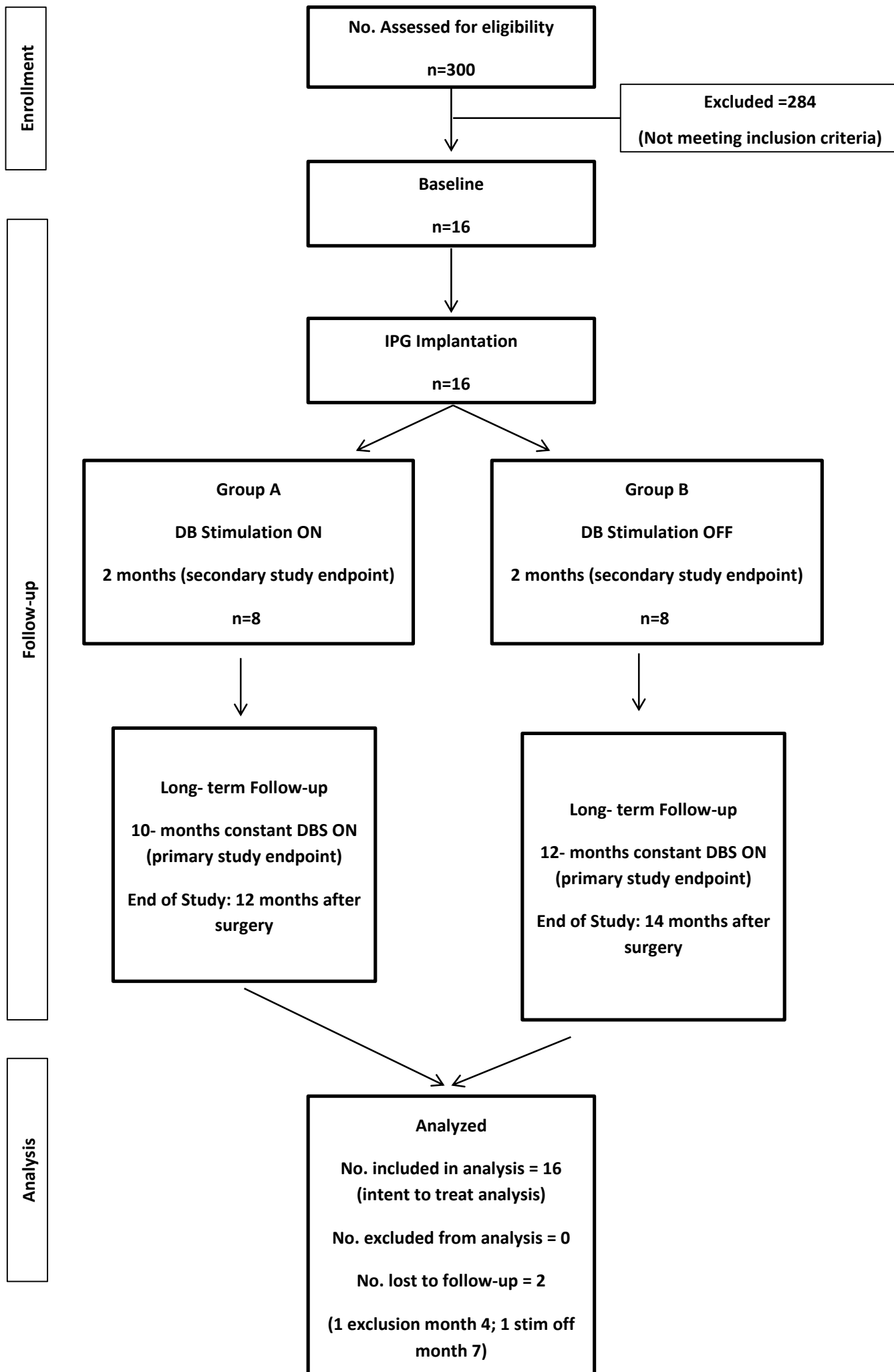

Supplement: Supplementary file 2 — CONSORT Flowchart [file 41386_2019_369_MOESM2_ESM.pdf]
