# Supplementary material for: Superolateral medial forebrain bundle deep brain stimulation in major depression: a gateway trial
Source: Neuropsychopharmacology. 2019 Mar 13;44(7):1224–32. doi: 10.1038/s41386-019-0369-9 (PMC6785007; doi:10.1038/s41386-019-0369-9)
Supplement: Supplementary file 3 — eTable 1 [file 41386_2019_369_MOESM3_ESM.docx]

**eTable 1. Neuropsychological assessment at baseline and eight Weeks after surgery for each group**

|  | |  | **GROUP A** |  | **GROUP B** |  |  |  |  |  |  |
| --- | --- | --- | --- | --- | --- | --- | --- | --- | --- | --- | --- |
| **Cognitive Domain** | |  | **Mean** | **SD** | **Mean** | **SD** | **Mean Change** | **SD Change** | ***t* Value** | ***df*** | ***p* Value** |
| **Verbal learning and memory** | | |  |  |  |  |  |  |  |  |  |
| VLMT total learning | | Baseline | 86.00 | 16.55 | 95.25 | 9.19 | -9.25 | 13.38 | -1.38 | 14 | 0.19 |
|  |  | 8 Weeks | 89.25 | 18.66 | 96.75 | 6.84 | -7.50 | 14.06 | -1.07 | 14 | 0.30 |
| **General cognitive functions** | | |  |  |  |  |  |  |  |  |  |
| MMSE (sum score) | | Baseline | 28.25 | 1.28 | 28.500 | 1.07 | -0.25 | 1.18 | -0.42 | 14 | 0.68 |
|  |  | 8 Weeks | 28.38 | 3.46 | 28.25 | 1.67 | 0.13 | 2.71 | 0.09 | 14 | 0.93 |
| MWT-B | | Baseline | 101.9 | 8.89 | 105.9 | 12.96 | -4.00 | 11.11 | -0.72 | 14 | 0.48 |
|  |  | 8 weeks | 102.0 | 9.93 | 110.5 | 11.69 | -8.50 | 10.84 | -1.57 | 14 | 0.14 |
| **Language** | | |  |  |  |  |  |  |  |  |  |
| HAWIE lexis test | | Baseline | 97.63 | 12.28 | 102.3 | 11.01 | -4.63 | 11.67 | -0.79 | 14 | 0.44 |
|  |  | 8 Weeks | 96.75 | 13.63 | 106.6 | 11.86 | -9.88 | 12.77 | -1.55 | 14 | 0.144 |
| HAWIE finding similarities | | Baseline | 101.6 | 8.23 | 104.1 | 10.21 | -2.50 | 9.28 | -0.54 | 14 | 0.60 |
|  |  | 8 Weeks | 101.3 | 13.41 | 109.3 | 9.72 | -8.00 | 11.71 | -1.37 | 14 | 0.19 |
| Word fluency | | Baseline | 95.43 | 10.53 | 91.88 | 6.90 | 3.55 | 8.82 | 0.78 | 13 | 0.45 |
|  |  | 8 Weeks | 86.71 | 7.09 | 86.13 | 10.41 | 0.59 | 9.03 | 0.13 | 13 | 0.90 |
| **Working memory** | | |  |  |  |  |  |  |  |  |  |
| Wechsler digit span | | Baseline | 104.3 | 12.01 | 104.4 | 9.18 | -0.13 | 10.69 | -0.02 | 14 | 0.98 |
|  |  | 8 Weeks | 106.1 | 15.24 | 99.75 | 9.01 | 6.39 | 12.30 | 1.00 | 13 | 0.33 |
| Wechsler vis. mem. span | | Baseline | 93.29 | 7.83 | 89.75 | 10.93 | 3.54 | 9.62 | 0.71 | 13 | 0.49 |
|  |  | 8 Weeks | 95.29 | 11.01 | 91.38 | 9.50 | 3.91 | 10.23 | 0.74 | 13 | 0.47 |

Note. Group A (active, DBS on), Group B (sham, DBS off); VLMT, Verbal Learning and Memory Test; MMSE, Mini-Mental State Examination; MWT-B, Multiple Choice Vocabulary Intelligence Test; HAWIE, Hamburg-Wechsler-Intelligenztest für Erwachsene; Wechsler vis. Mem. Span, Wechsler Visual Memory Span; TMT, Trail Making Test; Rey CFT, Rey Complex Figure Test ;STROOP int., Stroop Color and Word Test interference; RVDLT, Rey Visual Design Learning Test; VOT, Hooper Visual Organization Test; D2, Attention-Burden Test. Changes in cognitive performance between baseline and one year were analyzed via paired *t* tests for dependent samples for each neuropsychological test.

| **eTable 1 continued** | | | | | | | | | | | |
| --- | --- | --- | --- | --- | --- | --- | --- | --- | --- | --- | --- |
|  | |  | **GROUP A** |  | **GROUP B** |  |  |  |  |  |  |
| **Cognitive Domain** | |  | **Mean** | **SD** | **Mean** | **SD** | **Mean Change** | **SD Change** | ***t* Value** | ***df*** | ***p* Value** |
| TAP working memory | | Baseline | 97.57 | 8.34 | 102.2 | 9.45 | -4.60 | 8.87 | -0.93 | 11 | 0.37 |
|  |  | 8 Weeks | 93.00 | 8.93 | 92.86 | 10.70 | 0.14 | 9.85 | 0.03 | 12 | 0.98 |
| **Executive functions** | | |  |  |  |  |  |  |  |  |  |
| TMT | | Baseline | 89.38 | 12.50 | 92.50 | 10.56 | -3.13 | 11.58 | -0.54 | 14 | 0.60 |
|  |  | 8 weeks | 87.25 | 9.04 | 90.75 | 11.41 | -3.50 | 10.29 | -0.68 | 14 | 0.51 |
|  | |  |  |  |  |  |  |  |  |  |  |
|  | |  |  |  |  |  |  |  |  |  |  |
| Rey CFT Complex Figure Test | | Baseline | 93.88 | 13.30 | 93.50 | 18.21 | 0.38 | 15.95 | 0.05 | 14 | 0.96 |
|  |  | 8 weeks | 95.13 | 17.22 | 99.88 | 16.99 | -4.75 | 17.10 | -0.56 | 14 | 0.59 |
| STROOP int. (sec) | | Baseline | 94.13 | 7.16 | 100.1 | 8.04 | -6.00 | 7.60 | -1.58 | 14 | 0.14 |
|  |  | 8 Weeks | 91.75 | 10.19 | 96.75 | -5.00 | 9.52 | -1.05 | 14 | 0.31 |  |
| Five-Point Test | | Baseline | 102.8 | 5.68 | 103.3 | 9.39 | -0.50 | 7.76 | -0.13 | 14 | 0.90 |
|  |  | 8 Weeks | 103.0 | 6.07 | 102.9 | 7.30 | 0.13 | 6.71 | 0.04 | 14 | 0.97 |
| TAP go-nogo | | Baseline | 88.14 | 6.69 | 89.00 | 2.83 | -0.86 | 5.30 | -0.29 | 11 | 0.78 |
|  |  | 8 Weeks | 90.29 | 8.04 | 87.00 | 6.98 | 3.29 | 7.52 | 0.82 | 12 | 0.43 |
| **Visual spatial learning and memory** | | |  |  |  |  |  |  |  |  |  |
| RVDLT total learning | | Baseline | 86.75 | 11.24 | 93.87 | 15.34 | -7.13 | 13.45 | -1.06 | 14 | 0.31 |
|  |  | 8 Weeks | 85.62 | 12.95 | 92.75 | 20.70 | -7.13 | 17.26 | -0.83 | 14 | 0.42 |
| **Visual perception** | | |  |  |  |  |  |  |  |  |  |
| VOT | | Baseline | 99.13 | 12.17 | 106.4 | 11.56 | -7.25 | 11.87 | -1.22 | 14 | 0.24 |
|  |  | 8 Weeks | 101.8 | 12.91 | 107.9 | 11.04 | -6.13 | 12.01 | -1.02 | 14 | 0.33 |

Note. Group A (active, DBS on), Group B (sham, DBS off); VLMT, Verbal Learning and Memory Test; MMSE, Mini-Mental State Examination; MWT-B, Multiple Choice Vocabulary Intelligence Test; HAWIE, Hamburg-Wechsler-Intelligenztest für Erwachsene; Wechsler vis. Mem. Span, Wechsler Visual Memory Span; TMT, Trail Making Test; Rey CFT, Rey Complex Figure Test ;STROOP int., Stroop Color and Word Test interference; RVDLT, Rey Visual Design Learning Test; VOT, Hooper Visual Organization Test; D2, Attention-Burden Test. Changes in cognitive performance between baseline and one year were analyzed via paired *t* tests for dependent samples for each neuropsychological test.

| **eTable 1 continued** | | |  |  |  |  |  |  |  |  |  |
| --- | --- | --- | --- | --- | --- | --- | --- | --- | --- | --- | --- |
|  | | | **GROUP A** |  | **GROUP B** |  |  |  |  |  |  |
| **Cognitive Domain** | | | **Mean** | **SD** | **Mean** | **SD** | **Mean Change** | **SD Change** | ***t* Value** | ***df*** | ***p* Value** |
| **Attention** | | |  |  |  |  |  |  |  |  |  |
| D2 total minus error | | Baseline e | 81.25 | 7.13 | 91.13 | 14.26 | -9.88 | 11.27 | -1.75 | 14 | 0.10 |
|  |  | 8 Weeks | 83.25 | 6.41 | 86.00 | 13.22 | -1.75 | 10.39 | -0.34 | 14 | 0.74 |
| TAP altertness | | Baseline | 87-29 | 9.93 | 90.50 | 6.35 | -3.21 | 8.49 | -0.68 | 11 | 0.51 |
|  |  | 8 Weeks | 87.57 | 7.04 | 87.88 | 6.45 | -0.30 | 6.73 | -0.09 | 13 | 0.93 |
| TAP divided attention | | Baseline | 92.14 | 10.24 | 95.50 | 10.27 | -3.36 | 10.25 | -0.59 | 11 | 0.57 |
|  |  | 8 Weeks | 93.57 | 8.06 | 90.71 | 9.74 | 2.86 | 8.94 | 0.60 | 12 | 0.56 |

Note. Group A (active, DBS on), Group B (sham, DBS off); VLMT, Verbal Learning and Memory Test; MMSE, Mini-Mental State Examination; MWT-B, Multiple Choice Vocabulary Intelligence Test; HAWIE, Hamburg-Wechsler-Intelligenztest für Erwachsene; Wechsler vis. Mem. Span, Wechsler Visual Memory Span; TMT, Trail Making Test; Rey CFT, Rey Complex Figure Test ;STROOP int., Stroop Color and Word Test interference; RVDLT, Rey Visual Design Learning Test; VOT, Hooper Visual Organization Test; D2, Attention-Burden Test. Changes in cognitive performance between baseline and one year were analyzed via paired *t* tests for dependent samples for each neuropsychological test.
