# Supplementary material for: Superolateral medial forebrain bundle deep brain stimulation in major depression: a gateway trial
Source: Neuropsychopharmacology. 2019 Mar 13;44(7):1224–32. doi: 10.1038/s41386-019-0369-9 (PMC6785007; doi:10.1038/s41386-019-0369-9)
Supplement: Supplementary file 4 — eTable 2 [file 41386_2019_369_MOESM4_ESM.docx]

**eTable 2. Neuropsychological assessment at 6 months and 1 year DBS stimulation compared to baseline**

| **Cognitive Domain** | |  | **Mean change** | **SD change** | **n** | ***p* Value** |
| --- | --- | --- | --- | --- | --- | --- |
| **Verbal learning and memory** | | |  |  |  |  |
| VLMT total learning | | 6 months | 4.69 | 11.71 | 13 | 0.17 |
|  |  | 12 months | 10.15 | 12.90 | 13 | 0.02* |
| **General cognitive functions** | | |  |  |  |  |
| MMSE (sum score) | | 6 months | 0.31 | 1.70 | 13 | 0.53 |
|  |  | 12 months | 0.62 | 1.56 | 13 | 0.18 |
| MWT-B | | 6 months | 2.83 | 6.82 | 12 | 0.18 |
|  |  | 12 months | 5.23 | 5.10 | 13 | 0.00** |
| **Language** | | |  |  |  |  |
| HAWIE lexis test | | 6 months | 0.54 | 6.02 | 13 | 0.75 |
|  |  | 12 months | 2.15 | 5.84 | 13 | 0.21 |
| HAWIE finding similarities | | 6 months | 2.15 | 8.96 | 13 | 0.40 |
|  |  | 12 months | 4.69 | 7.79 | 13 | 0.05 |
| Word fluency | | 6 months | -2.75 | 7.61 | 12 | 0.24 |
|  |  | 12 months | -2.38 | 7.81 | 13 | 0.29 |
| **Working memory** | | |  |  |  |  |
| Wechsler digit span | | 6 months | -3.44 | 11.19 | 9 | 0.38 |
|  |  | 12 months | -3.38 | 9.52 | 13 | 0.22 |
| Wechsler vis. mem. span | | 6 months | -0.63 | 8.60 | 8 | 0.84 |
|  |  | 12 months | -0.83 | 13.55 | 12 | 0.84 |
| TAP working memory | | 6 months | -7.00 | 10.82 | 3 | 0.38 |
|  |  | 12 months | -5.67 | 13.50 | 3 | 0.54 |

Note. VLMT, Verbal Learning and Memory Test; MMSE, Mini-Mental State Examination; MWT-B, Multiple

Choice Vocabulary Intelligence Test; HAWIE, Hamburg-Wechsler-Intelligenztest für Erwachsene; Wechsler vis.

Mem. Span, Wechsler Visual Memory Span; Changes in cognitive performance between baseline six and

12 months were analyzed with paired *t* tests for dependent samples for each neuropsychological test.

Mean, mean change from baseline value, SD change, Standard deviation change from baseline value.

| **eTable 1 continued** | |  |  |  |  |  |
| --- | --- | --- | --- | --- | --- | --- |
| **Cognitive Domain** | |  | **Mean change** | **SD change** | **n** | ***p* Value** |
| **Executive functions** | |  |  |  |  |  |
| TMT | | 6 months | 2.50 | 10.42 | 10 | 0.46 |
|  |  | 12 months | 4.38 | 10.60 | 13 | 0.16 |
| Rey CFT | | 6 months | 6.54 | 15.69 | 13 | 0.16 |
|  |  | 12 months | 7.15 | 12.65 | 13 | 0.06 |
| STROOP int. (sec) | | 6 months | -0.20 | 8.24 | 10 | 0.94 |
|  |  | 12 months | 1.15 | 6.95 | 13 | 0.56 |
| Five-Point Test | | 6 months | 2.27 | 6.69 | 11 | 0.29 |
|  |  | 12 months | 0.08 | 12.81 | 12 | 0.98 |
| TAP go-nogo | | 6 months | -4.33 | 5.50 | 3 | 0.31 |
|  |  | 12 months | 2.67 | 8.02 | 3 | 0.62 |
| **Visual spatial learning and memory** | | |  |  |  |  |
| RVDLT total learning | | 6 months | -2.78 | 14.42 | 9 | 0.58 |
|  |  | 12 months | 0.23 | 9.50 | 13 | 0.93 |
| **Visual perception** | | |  |  |  |  |
| VOT | | 6 months | 2.8 | 5.47 | 10 | 0.14 |
|  |  | 12 months | 1.84 | 7.79 | 13 | 0.41 |

Note. TMT, Trail Making Test; Rey CFT, Rey Complex Figure Test; STROOP int., Stroop Color and Word Test

interference; RVDLT, Rey Visual Design Learning Test; VOT, Hooper Visual Organization Test;

Changes in cognitive performance between baseline six and 12 months were analyzed with paired *t* tests for

dependent samples for each neuropsychological test. Mean, mean change from baseline value, SD change,

Standard deviation change from baseline value.

| **eTable 1 continued** | |  |  |  |  |  |
| --- | --- | --- | --- | --- | --- | --- |
| **Cognitive Domain** | |  | **Mean change** | **SD change** | **n** | ***p* Value** |
| **Attention** | |  |  |  |  |  |
| D2 total minus error | | 6 months | 5.50 | 10.90 | 10 | 0.14 |
|  |  | 12 months | 6.08 | 8.85 | 12 | 0.06 |
| TAP altertness | | 6 months | -4.5 | 4.95 | 2 | 0.42 |
|  |  | 12 months | 2.00 | 10.39 | 3 | 0.77 |
| TAP divided attention | | 6 months | -4.0 | 7.55 | 3 | 0.46 |
|  |  | 12 months | -1.67 | 11.59 | 3 | 0.83 |

Note. D2, Attention-Burden Test. Changes in cognitive performance between baseline six and 12 months were

analyzed with paired *t* tests for dependent samples for each neuropsychological test. Mean, mean change from

baseline value, SD change, Standard deviation change from baseline value.
