# Supplementary material for: Superolateral medial forebrain bundle deep brain stimulation in major depression: a gateway trial
Source: Neuropsychopharmacology. 2019 Mar 13;44(7):1224–32. doi: 10.1038/s41386-019-0369-9 (PMC6785007; doi:10.1038/s41386-019-0369-9)
Supplement: Supplementary file 5 — eTable 3 [file 41386_2019_369_MOESM5_ESM.pdf]

eTable 3: Clinical Measures for sham and active DB stimulation

		Group A - active		Group B - sham		GLMM Statistics		
		Mean reduction	SD	Mean reduction	SD	Factor	t value	p-value
MADRS	Week 2	-18.38	5.48	-17.50	4.24	GROUP (on vs. off)	0.50	0.61
	Week 3	-17.50	8.67	-14.88	8.35	TIME (week 1-8)	4.37	<0.001
	Week 4	-18.50	7.63	-13.86	6.57	GROUPxTIME	-2.82	0.006
	Week 5	-14.50	8.78	-11.25	7.32			
	Week 6	-16.38	8.98	-9.88	6.38			
	Week 7	-18.57	11.24	-6.86	6.64			
	Week 8	-17.14	10.09	-8.14	9.12			
HDRS	Week 2	-16.50	6.32	-16.75	3.06	GROUP (on vs. off)	0.72	0.47
	Week 3	-15.63	8.98	-14.88	6.29	TIME (week 1-8)	3.27	0.002
	Week 4	-17.13	9.91	-15.00	7.44	GROUPxTIME	-2.23	0.03
	Week 5	-13.00	7.37	-11.13	7.51			
	Week 6	-13.88	8.84	-11.13	6.42			
	Week 7	-15.86	9.70	-9.57	8.89			
	Week 8	-16.43	9.41	-10.43	11.12			
SF-36ph	Week 2	0.33	13.11	0.13	7.88	GROUP (on vs. off)	-0.18	0.86
	Week 3	-2.33	5.99	-1.12	7.94	TIME (week 1-8)	-0.39	0.69
	Week 4	-2.00	8.72	-4.43	9.86	GROUPxTIME	0.48	0.63
	Week 5	-1.75	6.39	-3.71	7.57			
	Week 6	-0.86	8.05	-1.71	8.98			
	Week 7	-1.00	6.63	-1.14	11.03			
	Week 8	-3.29	4.75	0.57	8.99			
SF-36mh	Week 2	6.83	5.78	9.13	7.51	GROUP (on vs. off)	-0.36	0.72
	Week 3	9.00	7.18	7.50	5.83	TIME (week 1-8)	0.31	0.76
	Week 4	9.29	8.46	8.43	3.82	GROUPxTIME	-0.15	0.88
	Week 5	5.38	8.09	9.29	7.23			
	Week 6	6.86	11.91	11.57	7.98			
	Week 7	8.29	9.69	9.86	10.29			
	Week 8	8.43	11.54	6.86	11.23			
GAF	Week 2	14.88	8.48	17.00	12.05	GROUP (on vs. off)	-0.83	0.41
	Week 3	17.38	10.18	24.38	16.85	TIME (week 1-8)	-1.44	0.15
	Week 4	21.13	12.48	16.57	9.80	GROUPxTIME	1.07	0.29
	Week 5	14.75	8.96	19.13	11.96			
	Week 6	19.88	8.01	17.50	14.89			
	Week 7	16.86	11.14	16.43	15.26			
	Week 8	14.71	11.99	15.57	17.25			
BDI	Week 2	-16.00	13.03	-12.12	8.74	GROUP (on vs. off)	0.31	0.75
	Week 3	-12.83	9.68	-12.12	8.21	TIME (week 1-8)	0.09	0.93
	Week 4	-6.57	12.79	-12.28	6.72	GROUPxTIME	0.34	0.74
	Week 5	-7.37	8.79	-13.50	7.23			
	Week 6	-8.00	12.32	-14.25	6.06			
	Week 7	-13.42	12.42	-13.00	9.83			
	Week 8	-12.57	9.44	-12.00	9.38			
IDSSR	Week 2	-17.66	13.45	-16.00	12.23	GROUP (on vs. off)	0.94	0.34
	Week 3	-15.16	9.98	-16.62	14.21	TIME (week 1-8)	1.3	0.19
	Week 4	-8.42	10.53	-15.28	15.85	GROUPxTIME	-0.89	0.37
	Week 5	-9.50	9.30	-16.12	15.15			
	Week 6	-10.87	14.57	-16.37	14.04			
	Week 7	-16.85	11.79	-14.57	16.37			
	Week 8	-16.57	13.15	-12.57	13.80			
SLC-90	Week 2	-10.33	11.36	-16.85	8.37	GROUP (on vs. off)	1.15	0.25
	Week 3	-9.66	9.45	-13.75	11.74	TIME (week 1-8)	1.96	0.054
	Week 4	-2.80	10.68	-12.00	9.50	GROUPxTIME	0.58	0.56
	Week 5	-4.71	8.67	-15.87	9.62			
	Week 6	-6.85	9.88	-13.25	10.95			
	Week 7	-5.60	7.60	-11.42	11.78			
	Week 8	-7.16	7.88	-10.57	10.79			

Note. Clinical measures are analyzed with a MIXED Model between sham and active DBS groups from week 2 to week 8.
